# Chimeric antigen receptor T-cell therapy in autoimmune diseases

**DOI:** 10.3389/fimmu.2024.1492552

**Published:** 2024-11-19

**Authors:** Jie Liu, Yan Zhao, Hai Zhao

**Affiliations:** ^1^ Department of Neurosurgery, the Affiliated Hospital of Qingdao University, Qingdao, Shandong, China; ^2^ Department of Respiratory, Lanzhou University Second Hospital, Lanzhou, Gansu, China

**Keywords:** chimeric antigen receptors, autoimmune diseases, B cells, CD19, tolerance induction

## Abstract

The administration of T cells that have been modified to carry chimeric antigen receptors (CARs) aimed at B cells has been an effective strategy in treating B cell malignancies. This breakthrough has spurred the creation of CAR T cells intended to specifically reduce or alter the faulty immune responses associated with autoimmune disorders. Early positive outcomes from clinical trials involving CAR T cells that target the B cell protein CD19 in patients suffering from autoimmune diseases driven by B cells have been reported. Additional strategies are being developed to broaden the use of CAR T cell therapy and enhance its safety in autoimmune conditions. These include employing chimeric autoantireceptors (CAAR) to specifically eliminate B cells that are reactive to autoantigens, and using regulatory T cells (Tregs) engineered to carry antigen-specific CARs for precise immune modulation. This discussion emphasizes key factors such as choosing the right target cell groups, designing CAR constructs, defining tolerable side effects, and achieving a lasting immune modification, all of which are critical for safely integrating CAR T cell therapy in treating autoimmune diseases.

## 1 Introduction

Chimeric antigen receptors (CARs) are engineered fusion proteins that combine an antigen-recognition domain with cell-activating domains, allowing T cells to target and act against specific antigens (1, 2). This technology has transformed the treatment of hematological cancers, especially through the use of CAR T cells targeting CD19 in B cell malignancies (3, 4) (**Table 1**). These treatments have been highly effective, achieving long-term remission in many patients. Encouraged by these outcomes, researchers are now applying CAR T cell therapy to autoimmune diseases, where B cells often play a critical role in disease progression (20, 21).

**Table 1:** Reported clinical trials of CAR-T cells in treatment of autoimmune diseases.

Autoimmune diseases	CAR-T target	Outcome	Side Effects	Year	Ref.
Refractory SLE	CD19	Clinical remission was attained; the SLE Disease Activity Index score dropped from 16 to 0, with no evidence of disease recurrence for up to 18 months.	No adverse events related to CAR-T therapy were observed.	2021	(5)
SLE	CD19, BCMA	Significant B cell depletion was achieved by Day 198 after infusion; ANA titers were undetectable by 37 weeks.	No severe adverse event occurred	2021	(6)
Refractory SSc	CD19	CAR-T cells were detectable in the patients’ peripheral blood for up to nine months.	No CRS, ICANS, or prolonged cytopenia were observed.	2022	(7)
Refractory SLE	CD19 or BCMA	CAR-T cells proliferated *in vivo*, resulting in significant depletion of B cells.	Patients experienced grade 1 CRS without any ICANS; varying degrees of hematologic toxicity were noted.	2022,2023	(8, 9)
Severe SSc	CD19	CAR-T cells proliferated *in vivo*, resulting in complete B cell depletion.	Low-grade CRS was observed with no ICANS detected.	2023	(10)
Sjögren’s syndrome	CD19	Patients achieved complete remission with total depletion of antinuclear antibodies observed.	Grade 2 CRS and grade 1 ICANS were observed.	2023	(11)
Refractory antisynthetase syndrome	CD19	Physical improvements noted, with increased muscle strength and endurance; significant decrease in anti-Jo-1 antibodies; major improvement in antisynthetase syndrome observed, with tumor inflammation scores of 87.5 at Day 46 and 98 at Day 180.	Increase in myalgia and creatine kinase observed in patients due to B cell killing and CAR-T cell activation-induced inflammation.	2023	(12)
Antisynthetase syndrome	CD19	No signs of myositis detected on MRI; laboratory parameters, including CD8+ T cells subsets, inflammatory cytokines, and serological muscle enzymes, were normalized.	No severe adverse event occurred.	2023	(13)
Refractory myasthenia gravis	CD19	Total B cell depletion achieved; protective IgG levels.	No adverse events associated with CAR-T therapy, including CRS, ICANS, or insufficient hematopoietic reconstitution, observed.	2023	(14)
Severe SLE, idiopathic inflammatory myositis, and SSc	CD19	DORIS remission achieved in all patients with SLE; ACR–EULAR major clinical response achieved in all patients with idiopathic inflammatory myositis; decrease in EUSTAR activity index score achieved in all patients with SSc.	No severe adverse event occurred.	2024	(15)
SLE and LN	CD19, BCMA	Symptom remission and MFR from SLE achieved in two out of 13 patients; renal function significantly improved in ten patients with LN within 3 months post infusion.	No severe adverse event occurred.	2024	(16)
Rapidly progressive LN from hemodialysis	CD19	Full autoantibodies depletion achieved; renal function improved; patients achieved dialysis-free, partial renal response 4 months post therapy.	Grade 1 CRS and malaise observed, with no long-term bone marrow toxicity or ICANS.	2024	(17)
MS	CD19	Grade 2 and 3 CTCAE observed in patients; increased walking distance reported by patients.	Grade 1 or no CRS observed in patients; no ICANS observed; no treatment-related toxicity observed up to Day 100	2024	(18)
SSc-associated interstitial lung disease	CD19	All examined elements of autoimmunity-related interstitial lung disease and lung fibrosis showed improvement.	No severe adverse event occurred.	2024	(19)

SSC, Systemic Sclerosis; MRI, magnetic resonance imaging; MFR, medication-free remission; LN, Lupus Nephritis; MS, Multiple Sclerosis CTCAE; Common Terminology Criteria for Adverse Events.

**Figure 1:**
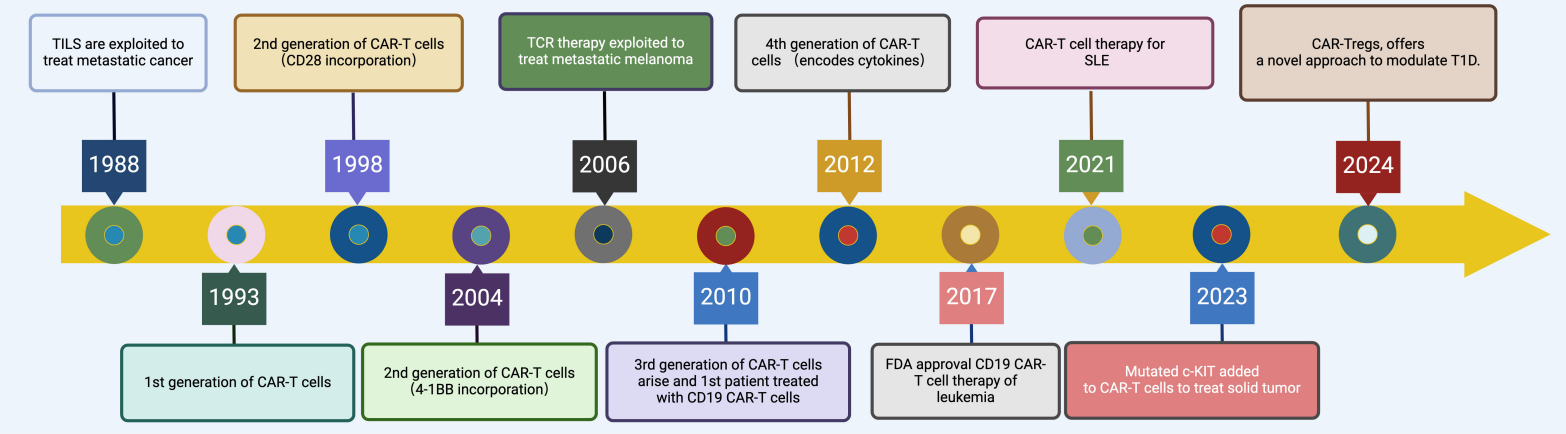
Evolution of CAR-T Cell Therapy. This timeline graphically depicts the evolution of CAR-T therapy from 1988 to 2024, highlighting key milestones such as the initial exploitation of TILs in 1988, the development of first-generation CAR-T cells in 1993, enhancements with second and third-generation CAR-T cells incorporating co-stimulatory molecules like CD28 and 4-1BB during the 1990s and 2010s, the groundbreaking FDA approval of CD19-targeted CAR-T therapy for leukemia in 2017, the extension of CAR-T therapies to autoimmune diseases like SLE in 2021, and the innovative adaptation to non-hematological malignancies and immunomodulatory diseases with mutated c-KIT targeted CAR-T cells and CAR-T regulatory cells for T1DM by 2024. Created in BioRender.com.

Early trials using anti-CD19 CAR T cells have shown promising results in treating conditions like SLE (systemic lupus erythematosus) and other autoimmune disorders (5, 10, 12, 22, 23). Efforts are also underway to develop CARs that target different pathogenic cells, such as plasma cells that produce antibodies, expanding the potential of CAR T cell therapy to modulate complex immune dysfunctions in autoimmune diseases (24). **Figure 1** illustrates the historical progression of CAR-T therapy, tracing key developmental milestones from the initial exploitation of tumor-infiltrating lymphocytes (TILs) for metastatic cancer treatment in 1988 to the innovative approaches such as CAR-Treg therapy for modulating type 1 diabetes mellitus (T1DM) projected by 2024 (**Figure 1**).

Autoimmune conditions are also characterized by a failure of immunological tolerance, often due to inadequate regulation by Tregs (25). Recent strategies focus on enhancing the specificity and effectiveness of Treg cells using CAR technology to direct them more accurately to sites of inflammation or against specific effector T cells (26–30).

**Figure 2:**
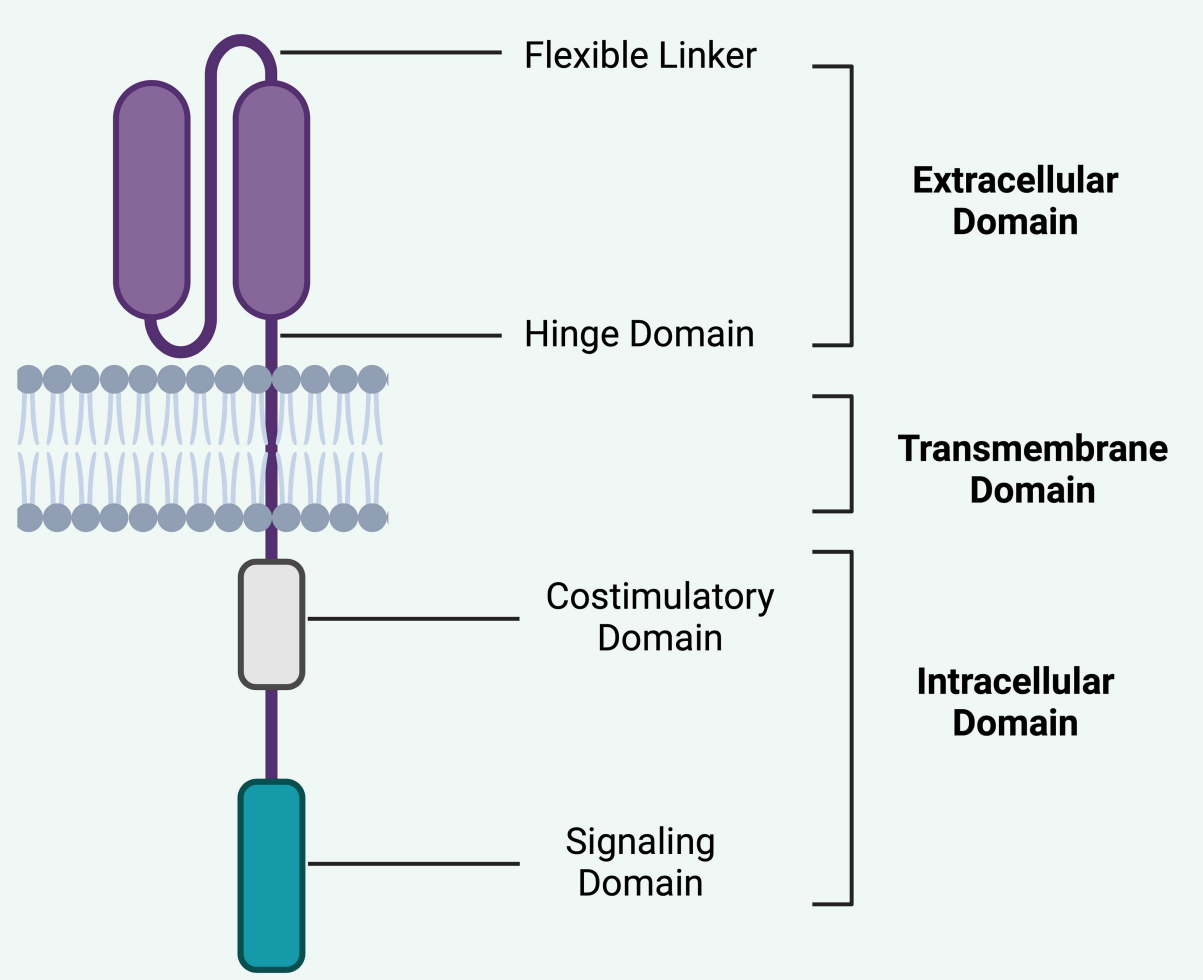
Basic structure of CAR. The structural components of a CAR is crucial in understanding its therapeutic efficacy and safety profile in CAR T-cell therapy. i).Extracellular domain typically consists of a scFv derived from an antibody. This domain is responsible for the specificity of the CAR, as it binds to a particular antigen on the target cells. The specificity is crucial for minimizing off-target effects, thereby enhancing the safety profile of the therapy by targeting only cancerous or diseased cells and sparing healthy tissues. ii). Hinge domain: This flexible linker connects the scFv to the transmembrane domain and provides spatial flexibility, allowing the extracellular domain to effectively engage with the target antigen. This flexibility can influence the ability of the CAR T-cells to bind to cells where antigens may be densely packed or in challenging structural configurations, potentially affecting both efficacy and safety by altering the activation threshold of the CAR T-cells. iii). Transmembrane domain anchors the CAR in the T-cell membrane and contributes to the stability of the receptor. The stability of this domain is important for the long-term persistence and survival of CAR T-cells, which are important for the sustained therapeutic effect but can also pose risks such as prolonged cytotoxic activity or CRS. iv). Costimulatory Domain segment is crucial for providing the necessary signals to fully activate the CAR T-cell upon antigen recognition. Common costimulatory domains include CD28 or 4-1BB. These domains can enhance the proliferation, survival, and overall antitumor function of CAR T-cells. The choice and combination of costimulatory domains can significantly affect the potency and potential side effects of the therapy, such as the risk of CRS or ICANS, by influencing the intensity and duration of T-cell activation. v). Intracellular Domain (Signaling Domain) typically involves CD3ζ, which initiates the signal transduction cascade leading to T-cell activation. This domain is critical for the function of CAR T-cells, as it translates the binding of the extracellular domain to an intracellular response, leading to T-cell proliferation, cytokine production, and cytotoxic activity. The design of this domain affects how robustly the CAR T-cells respond upon antigen engagement, which is central to both efficacy and safety, particularly regarding the risks of overactivation and associated toxicities. Each component of the CAR design directly impacts the balance between therapeutic efficacy and safety. By modifying these components, researchers can tailor CAR T-cell therapies to maximize therapy effect while minimizing the risks of severe side effects. This ongoing optimization continues to be a major focus in the development of CAR T-cell therapies, aiming to broaden their applicability and improve their safety profile. Created in BioRender.com.

However, CAR T cell therapy can lead to severe side effects, such as cytokine release syndrome (CRS) and immune effector cell-associated neurotoxicity syndrome (ICANS), which, while generally reversible, can be life-threatening. Interestingly, the application of CAR T cell therapy in autoimmune diseases tends to have a milder safety profile compared to oncology settings (31, 32) (**Figure 2**).

This review considers the selection of target cell populations, appropriate CAR constructs, acceptable levels of toxicity, the necessity of pre-conditioning regimens, and the potential to achieve lasting immune modulation or tolerance. The evolving research indicates that CAR T cell therapy could markedly change the treatment landscape for autoimmune diseases, pointing towards a new era of targeted immunotherapies.

## 2 Targeting B cells in autoimmune diseases

In certain autoimmune diseases, B cells are directly implicated in pathology by producing autoantibodies. While the role of autoantibodies in many autoimmune diseases remains less defined, it is clear that most patients with these conditions exhibit impaired B cell tolerance, leading to the survival of autoreactive B cells due to failures in both peripheral and central tolerance mechanisms (33). B cells also exacerbate disease through the production of inflammatory cytokines and acting as antigen-presenting cells (34). The presence of B cells in diseased tissues and the positive response to therapies targeting B cell antigens like CD20 and CD19 underscores their pivotal role in such diseases.

The mAb (monoclonal antibody) rituximab, initially approved in 1997 for refractory B cell non-Hodgkin lymphomas, has since proven effective in autoimmune diseases like rheumatoid arthritis and pemphigus vulgaris, targeting B cells through CD20 (35). Other mAbs targeting CD20, such as ocrelizumab and ofatumumab, have been authorized for B cell-related neurological autoimmune disorders, including MS (multiple sclerosis) (36). Additionally, belimumab targets the B cell survival factor BAFF (B-cell Activating Factor), showing efficacy in SLE and lupus nephritis by inhibiting B cell survival and differentiation into plasma cells (37).

Despite these advancements, some autoimmune conditions remain unresponsive or partially responsive to these therapies. For instance, abnormal B cell infiltration in tissues such as the renal interstitium in lupus nephritis contributes to ongoing disease activity, with studies showing persistent B cell presence even after rituximab treatment (38, 39). Anti-CD20 therapies deplete B cells through mechanisms like direct cell death, complement-dependent cytotoxicity, and antibody-dependent cellular cytotoxicity, with varying efficacies based on the antibody type (40, 41). However, challenges in effectively depleting tissue-resident B cells and the microenvironment’s impact on B cell survival pose significant obstacles (42).

Recent developments in anti-CD19 therapy show promise for broader B cell depletion, as CD19 is expressed across more B cell subsets than CD20, potentially impacting a wider array of pathogenic B cells (43). However, anti-CD19 therapies like inebilizumab face challenges in depleting bone marrow plasma cells, a crucial site for autoantibody production (44). Moreover, the efficacy of bispecific antibodies, which facilitate interactions between T cells and B cells, remains underexplored in autoimmune diseases compared to their use in oncology (45).

CAR T cell therapy targeting CD19 has demonstrated significant tissue-based B cell depletion, improving outcomes in autoimmune models such as lupus by reducing autoantibody levels and disease symptoms. While effective, this approach may not fully deplete LLPCs, which maintain humoral immunity against infections and vaccines but also continue to produce autoantibodies relevant to autoimmune pathology (19, 46).

Overall, the effectiveness of B cell-targeting therapies in autoimmune diseases depends not only on the depth of B cell depletion but also on the breadth across various B cell maturation stages. The continuous evolution of therapeutic strategies, particularly those involving advanced modalities like CAR T cells, holds significant promise for more comprehensive and effective treatments for autoimmune diseases.

## 3 CAR design

CARs are composed of five distinct domains that can each be tailored to enhance the functionality of CAR T cells (**Figure 2**). These include the antigen-recognition domain, typically a single-chain variable fragment (scFv) from a mAb that enables HLA-independent antigen targeting. This is connected through a hinge domain to a transmembrane domain. Inside the cell, a co-stimulatory domain (often derived from CD28 or 4-1BB) and a T cell activation domain (commonly from CD3ζ) are integral for the activation and proliferation of CAR T cells (47, 48). In both preclinical and clinical evaluations of CAR T cells, researchers have utilized prototypical sequences from diverse molecules within each segment. Typically, the antigen-binding domain comprises a scFv sourced from a mAb, such as those targeting human CD19 in FDA-approved CAR T cell therapies (49). The intracellular signaling domain usually incorporates a T-cell activation module from the CD3ζ component of the T cell receptor along with co-stimulatory domains that often include regions containing immunoreceptor tyrosine-based activation motifs from CD28 or 4-1BB (also referred to as CD137 and TNFRSF9) (50–52). Modifications to the CAR gene constructs allow for the engineering of CAR T cells that express an ‘armor’ protein, typically a cell-surface or secreted immunomodulatory molecule, which bolsters T cell efficacy or beneficially alters the tumor microenvironment (53). Alterations in these components of CAR constructs facilitate precise adjustments to the function and anti-tumor potency of the resultant CAR T cell products, with various CAR designs currently under development to enhance the safety and effectiveness of these treatments across multiple cancer types (54). Moreover, the genetic modification of these engineered T cells to augment CAR T cell functionality represents a burgeoning field of research.

Beyond this standard monospecific CAR structure, innovative designs are being explored to increase efficacy and reduce toxicity. For example, bicistronic CARs can target two antigens simultaneously, such as CD19 and B cell maturation antigen (BCMA), which could be more effective in eliminating autoimmunity-inducing antibodies by targeting both B cells and long-lived plasma cells (LLPCs) (55). Another variant, T cell receptor (TCR) fusion constructs or TRuCs, merge scFv domains with TCR subunits, potentially reducing cytokine release and thus minimizing clinical toxicity (56).

The “armored CAR” approach includes co-expressing another therapeutic protein, such as a cytokine, with the CAR construct to modulate the immune environment directly at the site of autoimmune activity (57). For example, combining an anti-CD19 CAR with IL-10 could mitigate inflammation in autoimmune diseases.

Upon antigen recognition, CAR T cells activate to release cytokines, exhibit cytotoxic effects, and proliferate. Ideally, CAR T cells should exclusively target cells expressing the antigen without cross-reactivity, which can lead to non-specific activation or off-target effects (58, 59). Challenges with CAR designs include non-specific antigen recognition, overactivation resulting in severe cytokine release, and continuous signaling that may lead to T cell exhaustion. Managing these issues, especially CRS and ICANS, is crucial in autoimmune therapies, considering the longer life expectancies of these patients compared to those with hematological malignancies (60). Each component of the CAR construct needs careful consideration to balance efficacy and safety, aiming for a therapeutic profile that maximizes patient benefit while minimizing adverse effects.

Furthermore, a CAAR incorporates pathogenic autoantigens within its antigen-recognition domain to specifically target autoantibodies associated with autoimmune diseases (61, 62). CAAR-T cells are an engineered variant of CAR-T cells designed to target and eliminate cells that produce pathogenic antibodies, such as autoreactive B cells (63, 64). The structure of CAAR-T cells incorporates a specific antigen tailored to recognize autoantibodies, a transmembrane domain, and an intracellular signaling domain, which may or may not include a co-stimulatory domain (65). These cells engage with and destroy autoreactive cells displaying the target autoantibodies through their engineered antigen specificity. A critical aspect in the development of CAAR-T cells is the careful selection and design of the specific antigen, which must be finely tailored to recognize and bind the corresponding autoantibodies accurately. Chimeric autoantigen T Cell Receptor (CATCR) cells represent a therapeutic strategy where T cells are engineered to express T-cell receptors that specifically target autoantigens—proteins or molecules mistakenly attacked by the immune system in autoimmune diseases. This approach is designed to target and modulate autoreactive T cells that recognize these autoantigens, which could help in re-establishing immune tolerance and reducing inflammation. As of the latest updates, CAAR T-cell therapy has been specifically explored in the treatment of pemphigus vulgaris, an autoimmune disease characterized by the presence of pathogenic autoantibodies that target skin cells (66). On the other hand, CATCR therapy remains largely experimental, with its potential applications discussed theoretically for autoimmune diseases such as T1DM, MS, and rheumatoid arthritis. Both therapies are in the early stages of research and development, focusing on highly specific targeting of disease-causing elements in the immune system to mitigate or potentially cure these complex conditions (67).

### 3.1 Antigen-recognition domain

The antigen-recognition domain is crucial in determining the sensitivity and specificity with which a CAR identifies its target antigen (47). This domain’s design is pivotal, especially when targeting malignancies, as it requires high affinity to be activated by minimal levels of tumor cell antigens. Its specificity must also be meticulously refined to avoid cross-reactivity with similar antigens on non-target cells, as such interactions could lead to severe adverse effects (68, 69).

There have been significant clinical repercussions due to cross-reactivity in oncological applications. For example, T cells targeting the melanoma-associated antigen 3 (MAGE-A3) mistakenly interacted with Titin, a protein prevalent in the heart, resulting in fatal cardiac toxicity in one instance (69). Another case saw MAGE-A3-targeted T cells react with a neuronal protein, causing fatal neurological outcomes (70). Additionally, a patient experienced fatal pulmonary toxicity from CAR T cells directed against the oncogenic protein ERBB2, illustrating the critical need for precision in the design of the antigen-recognition domain (71).

The primary targets for CAR T cell therapy in autoimmune diseases are CD19 and BCMA, where the risk of non-specific activity leading to severe tissue damage is comparatively lower, largely due to the extensive testing and application of these targets in oncology, confirming manageable toxicity levels (6, 72). However, the immunogenicity of CAR constructs poses another challenge, often stemming from their origin in human or mouse mAbs. This can provoke immune responses in patients against the foreign elements of the CAR, such as non-human antibody sequences or the synthetic linkers used in scFvs. In some instances, these immune responses have been shown to impair CAR T cell functionality by targeting the scFv itself (6, 73).

To mitigate these issues, CARs incorporating binding domains from human or humanized antibodies have been developed and are undergoing clinical trials (68, 74). Although these are hypothesized to be less immunogenic than their mouse-derived counterparts, conclusive evidence of their superiority remains pending. The context of autoimmune disease further complicates this, as patients may exhibit heightened immune responses to foreign proteins. For example, while anti-drug antibodies to rituximab—a chimeric anti-CD20 antibody—were infrequent in lymphoma patients, they were significantly more common in patients with systemic autoimmune diseases or SLE, highlighting the variability in immune reactivity across different patient groups (75–77).

The development of CARs for autoimmune conditions thus not only requires careful consideration of antigen specificity and immune compatibility but also a deeper understanding of the unique immunological landscapes of these diseases to optimize safety and efficacy.

### 3.2 Hinge and transmembrane domains

The hinge and transmembrane (HTM) domains of CARs play a significant role in influencing the overall function and safety profile of CAR T cells (48, 78). These domains not only support the structural integrity of the receptor but also affect its signaling dynamics, which can lead to variations in therapeutic and side effect profiles (59).

Research comparing anti-CD19 CARs differing only in their HTM domains—one derived from CD8α and the other from CD28—demonstrated that the choice of HTM domain can significantly impact cytokine release, a critical factor in the development of side effects such as CRS and neurological toxicities. *In vitro* studies showed that T cells equipped with the CD8α HTM domain released lower levels of toxic cytokines compared to those with the CD28 HTM domain. However, these differences in cytokine release did not translate to differences in anti-tumor efficacy in experimental models, as both constructs performed similarly in eliminating tumor cells in mice (59).

Clinical trials further illustrated the impact of HTM domain selection on safety profiles. An anti-CD19 CAR incorporating a human scFv and CD8α HTM domain (Hu19-CD828Z) was associated with a significantly lower incidence of neurological toxicity (5%) compared to an anti-CD19 CAR with a murine scFv and CD28 HTM domain (FMC63-28Z), which showed a much higher incidence (50%) (68). This reduced toxicity in the Hu19-CD828Z group also corresponded with lower serum levels of immune mediators like granzyme A and CC chemokine ligand 2, indicating a milder immune response (68).

Interestingly, despite these differences in side effect profiles, the therapeutic outcomes—specifically anti-lymphoma effects—were very similar between patients receiving either the Hu19-CD828Z or FMC63-28Z CAR T cells (68). This suggests that while HTM domains influence safety, they do not compromise the efficacy of the CAR T cells against cancer (49). In further studies, CAR T cells featuring a longer CD8α hinge domain showed no neurological toxicity and only mild CRS, yet maintained comparable anti-lymphoma activity to historical controls. This finding supports the notion that optimizing the design of HTM domains can enhance the safety of CAR T therapies without sacrificing their effectiveness (49).

These observations underscore the importance of carefully selecting and designing HTM domains in the development of CAR T cell therapies, not only to maintain high efficacy but also to minimize potential side effects, thereby improving the overall therapeutic index of these powerful immune therapies.

### 3.3 Co-stimulatory domain

The presence of co-stimulatory domains within CARs such as CD28 or 4-1BB significantly enhances their functionality and anti-tumor efficacy (47, 79, 80). These domains are crucial for improving T cell persistence, activation, and overall antitumor responses. Clinical outcomes for CAR T cells utilizing either CD28 or 4-1BB have shown similar efficacy in treating B cell lymphomas, with comparable lymphoma response rates and progression-free survival (81, 82). However, CARs incorporating the 4-1BB domain tend to demonstrate longer *in vivo* persistence compared to those with the CD28 domain, although direct clinical comparisons to substantiate this difference are lacking (47). The variation in persistence might be influenced by multiple factors, including different HTM domains or the methods used to measure CAR T cell levels in the blood (83).

For autoimmune diseases, the optimal choice of co-stimulatory domain is less clear. The primary consideration in selecting a co-stimulatory domain for CARs aimed at treating autoimmune conditions should focus on minimizing potential toxicity while maintaining therapeutic efficacy.

### 3.4 CD3ζ signaling domain

The CD3ζ domain is a critical component in almost all clinically utilized CAR T cells (21, 47, 48). It contains three immunoreceptor tyrosine-based activation motifs (ITAMs). The phosphorylation of these ITAMs is crucial for T cell activation. However, this activation process can also lead to apoptosis, presenting a balance that must be managed to maximize therapeutic efficacy while minimizing cell death (84).

Research indicates that inactivating two of the three ITAMs within the CD3ζ domain can enhance the survival of CAR T cells by reducing activation-induced cell death. This modification could potentially improve the safety and longevity of CAR T cells in clinical settings (84). Further innovations include exploring other CD3 chains beyond CD3ζ. For example, CARs incorporating the CD3δ chain have demonstrated improved anti-tumor activity and reduced cytokine release in experimental models, suggesting potential benefits in terms of enhanced efficacy and reduced toxicity (83).

For both autoimmune and cancer therapies, the design of CAR T cells continues to evolve, with modifications to co-stimulatory and signaling domains aimed at optimizing their therapeutic profiles. This ongoing development underscores the complexity and potential of CAR technology in treating a wide range of diseases by fine-tuning immune cell functions.

## 4 Method of CAR gene transfer

The method of gene transfer for CAR T cells plays a crucial role in the safety and efficacy of the therapy (85–87). In most clinical trials for autoimmune diseases, lentiviral vectors have been commonly used to transfer CAR genes into patient T cells (85). Rapamycin, an mTOR inhibitor, has demonstrated therapeutic potential in autoimmune diseases due to its immunomodulatory properties, including reducing T cell activation and stabilizing regulatory T cells (88–94). Recent studies show that rapamycin can enhance CAR gene delivery into T cells by reducing antiviral mechanisms, specifically downmodulating interferon-induced transmembrane proteins (IFITMs), which serve as restriction factors (87, 95). This effect not only improves gene delivery rates but also sustains CAR T-cell functionality without adverse impacts on cell viability or specificity (87). Given these findings, mTOR blockade via rapamycin may improve CAR T-cell therapy in autoimmune diseases by enhancing transduction efficiency and potentially reducing immune-related adverse events, thereby contributing to both therapeutic safety and efficacy.

However, one significant drawback of using lentiviral vectors is the risk of insertional mutagenesis, which can lead to the development of secondary cancers such as T cell lymphomas (85). Although rare, with occurrences in only 22 out of more than 27,000 treated individuals, the detection of the CAR transgene within malignant clones in three cases underscores the potential risks associated with this method of gene transfer (85).

## 5 Use of anti-CD19 CAR T cells to treat autoimmune disease

Given these concerns, researchers have been exploring more targeted approaches for gene insertion to mitigate these risks. One promising method involves using the CRISPR-Cas9 system to direct CAR gene insertion specifically to the TRAC locus, which encodes the T-cell receptor α constant region in T cells (96, 97). This targeted insertion aims to enhance safety by reducing the likelihood of insertional mutagenesis. However, clinical-scale application of this precise gene-editing technology is still under active investigation (19).

**Figure 3:**
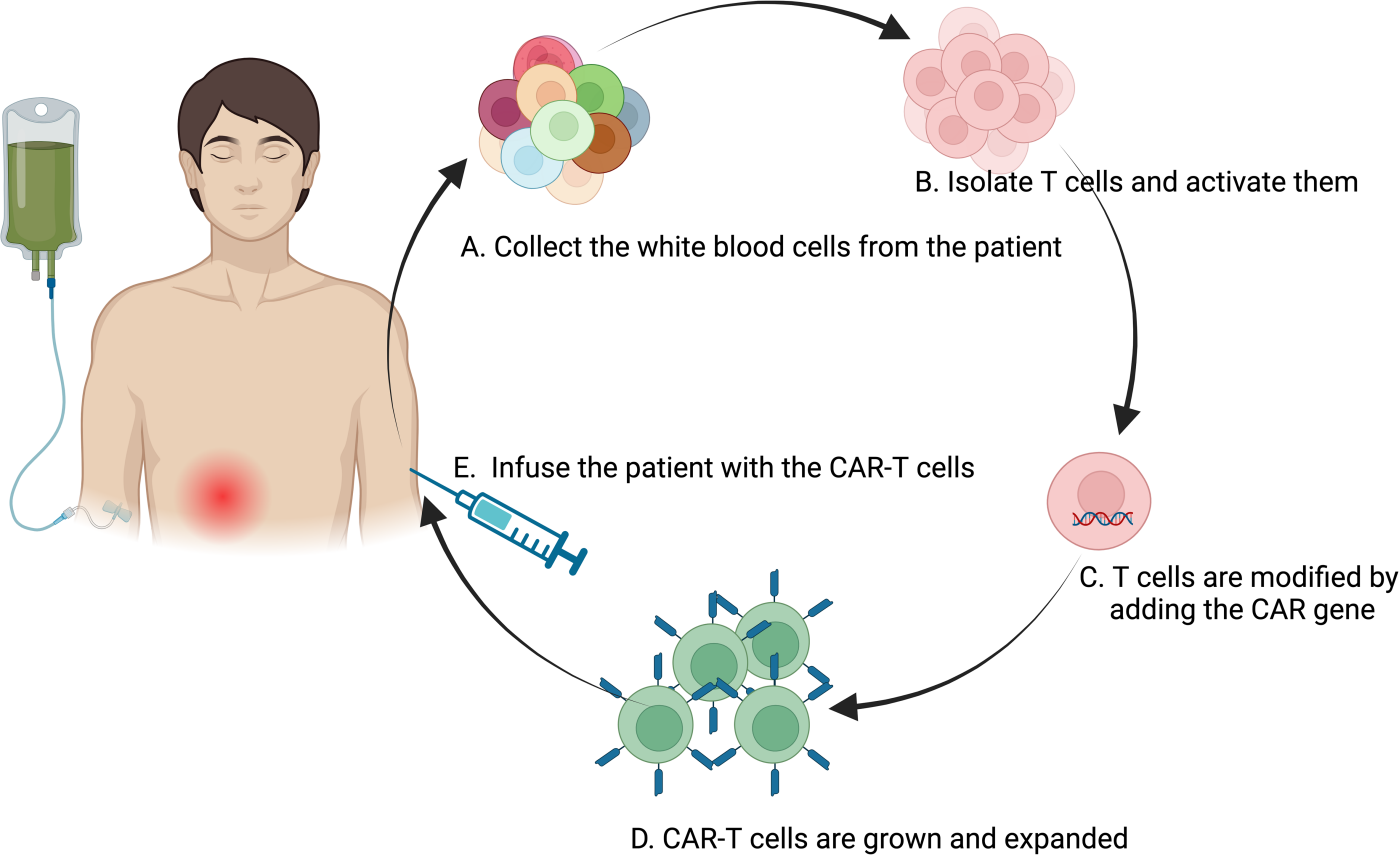
Flowchart of the CAR-T Cell Therapy Process. The process of CAR-T therapy involving several key steps: **(A)** Collection of white blood cells from the patient using a process similar to blood donation, typically through apheresis; **(B)** Isolation and activation of T cells from the extracted white blood cells; **(C)** Genetic modification of the activated T cells by introducing the CAR gene, programming the T cells to target and destroy cancer cells expressing a specific antigen; **(D)** Expansion of the engineered CAR-T cells in the laboratory to increase their numbers for an effective therapeutic dose; **(E)** Infusion of the engineered CAR-T cells back into the patient’s bloodstream, where they seek out and eliminate cancer cells. This representation encapsulates the sophisticated bioengineering involved in CAR-T therapy, highlighting its potential as a personalized treatment modality for cancer. Created in BioRender.com.

The utilization of anti-CD19 CAR T cells in treating autoimmune diseases offers a strong rationale due to their demonstrated ability to completely deplete B cells, including tissue-resident types (98)(**Table 1**). This capability has been effectively shown in conditions such as acute lymphoblastic leukemia and B cell lymphoma (98). Concerns that autoreactive CD4+ T cell clones might exacerbate the underlying autoimmune condition appear to be manageable, as studies in lupus mouse models using CAR T cells have effectively depleted B cells and ameliorated disease without enhancing autoimmune symptoms (99) (**Figure 3**).

In clinical settings, the use of unfractionated CD3+ lymphocytes to develop anti-CD19 CAR T cells has shown promising results without worsening disease symptoms. For instance, a notable case involved a 20-year-old patient with severe, treatment-resistant SLE, who responded dramatically well to autologous anti-CD19 CAR T cell therapy (5). The treatment led to a rapid resolution of lupus symptoms and was well tolerated, with no occurrences of CRS or ICANS (5). Other patients treated similarly experienced rapid remission and favorable safety profiles compared to those seen in oncology applications.

Despite these successes, not all autoantibodies were consistently reduced across patients, indicating that B cells might contribute to disease pathogenesis through other mechanisms, such as cytokine production or antigen presentation. Moreover, certain autoantibodies produced by CD19-negative LLPCs that are resistant to depletion by anti-CD19 CAR T cells suggest that additional strategies may be needed to target these resistant B cell subsets (100).

The efficacy of CAR T cell therapy across various autoimmune conditions, where patients had previously not responded to other B cell-targeting treatments, supports the potential of this therapy to achieve deep and broad B cell depletion (98, 101). Ongoing clinical trials continue to explore and validate the effectiveness of CAR T cell therapy in a wider spectrum of autoimmune diseases, promising a new horizon in the management and potential remission of these challenging conditions (7, 10, 12, 13, 102).

Anti-CD19 CAR T cell therapy, while transformative for cancer treatment, carries inherent risks when applied to autoimmune diseases, where the safety profile may differ due to the absence of tumor masses or malignant cell infiltration typically seen in cancers.

## 6 Safety considerations in CAR-T therapy

Safety considerations for CAR T cell therapy in autoimmune diseases are markedly distinct from those applicable to cancer therapy due to differences in the underlying health risks and expected outcomes (103). CAR-T cell therapy for cancer presents substantial risks due to its severe side effects, particularly CRS and immune effector cell-associated neurotoxicity syndrome (ICANS). For instance, in a study where CD19-targeting CAR-T cells were used to treat refractory large B cell lymphoma, severe CRS (grade >3) was reported in 13% of participants, leading to fatalities in two cases, while neurotoxicity was observed in 28% of the patients. Additionally, long-lasting adverse effects were noted, including continuous B cell aplasia affecting 25-38% of patients, a significant reduction in immunoglobulin levels seen in 18-74% of patients, and chronic cytopenia persisting for more than three months (104). Autoimmune disease patients generally exhibit significantly lower mortality rates compared to those suffering from relapsed or refractory B cell malignancies. Clinical trials indicate that the majority of autoimmune disease patients receiving CAR-T cell therapy typically report few to no adverse effects, such as CRS, ICANS and extended cytopenia (5, 7, 8, 14). From a causative perspective, CAR-T cell therapy in oncology is effective due to the abundance of antigen-expressing tumor cells that promote CAR-T cell proliferation and often lead to complete remission in high tumor burden scenarios. However, this potent response can trigger serious complications complicating the management of dosages. In contrast, autoimmune diseases generally demonstrate a safer profile for CAR-T cell therapy. This safety is attributed to the typically lower number of target B cells in autoimmune conditions compared to the vast numbers found in malignant conditions like leukemia or lymphoma (**Table 1**). However, the variation in the number of patients should also be factored into the evaluation of the safety profile.

Long-term B cell depletion in oncology patients treated with anti-CD19 CAR T cells often leads to hypogammaglobulinemia (105, 106). However, protective antibody titers, such as those against common pathogens and vaccines, generally remain stable. Similarly, in autoimmune patients treated with CAR T cells, immunoglobulin levels, including IgG, and vaccine-induced antibodies have not shown significant declines, which may be attributed to the survival of CD19-negative LLPCs that are not targeted by the therapy (14, 22).

Post-therapy infections are a noted complication in oncology patients treated with CAR T cells, with severe infections occurring in a significant minority (81, 107–109). Additionally, patients with persistent B cell aplasia are at a heightened risk of severe outcomes from COVID-19 (Coronavirus disease 2019) and may respond poorly to COVID-19 vaccinations (110, 111). However, in autoimmune disease contexts, the transient nature of B cell depletion and the potential for quicker B cell recovery may mitigate these risks. Initial findings suggest that the infection risk post-treatment may be lower in autoimmune patients, likely due to their less aggressive cytotoxic treatment histories and the ability to more quickly replenish B cells (10, 12, 22).

Despite some less severe aspects, there is still a need for enhanced safety measures and tailored therapeutic strategies to further reduce the risk of adverse effects associated with this therapy. This might involve the development of refined CAR-T constructs designed to decrease the potential for CRS and neurotoxicity, or the implementation of pre-treatment and monitoring protocols that are specifically adjusted to the sensitivities and medical backgrounds of autoimmune disease patients. Such strategies aim to maximize the therapeutic benefits of CAR-T cell therapy while minimizing its risks, ensuring a higher standard of care for patients with autoimmune diseases.

## 7 Targeting long-lived plasma cells

Targeting LLPCs is a pivotal strategy in the management of autoimmune diseases where these cells are major contributors to the production of pathogenic autoantibodies (111). Here’s a detailed look at how LLPCs are approached in therapy, with a focus on their implications in SLE and other autoimmune conditions:

### 7.1 Efficacy of targeting LLPCs

In patients with SLE, treatments using anti-CD19 CAR T cell therapy have shown rapid reductions in anti-double-stranded DNA antibodies, suggesting that CD19-positive plasmablasts and plasma cells are primary sources of these autoantibodies (98). However, the role of CD19-negative LLPCs, which can escape depletion by anti-CD19-based therapies, is still under investigation, particularly in SLE and other specific autoimmune diseases.

### 7.2 Approaches to deplete plasma cells

Daratumumab: This is a mAb targeting CD38, a marker overexpressed in plasma cells, particularly in multiple myeloma (112, 113). Daratumumab facilitates the depletion of plasma cells through mechanisms like antibody-dependent cellular cytotoxicity and complement-dependent cytotoxicity (114). It has been adapted for use in autoimmune diseases, demonstrating efficacy in reducing autoantibody levels produced by LLPCs (96).

Bortezomib: As a proteasome inhibitor, bortezomib induces apoptosis in plasma cells by disrupting the proteasome pathway, which is crucial for clearing misfolded proteins that cause cellular stress. This approach has been effective in autoimmune conditions by lowering the survival of LLPCs and thus reducing autoantibody levels (115).

The use of therapies like daratumumab and bortezomib in autoimmune diseases has been associated with broad depletion of bone marrow LLPCs. This results in significant reductions in serum IgG levels and the titers of protective antibodies against previous infections and vaccinations (116). Such outcomes necessitate a careful balance between reducing pathogenic autoantibodies and maintaining sufficient immunity against pathogens.

While initial reports suggest clinical benefits from targeting BCMA, ongoing assessments must consider the long-term infectious risks associated with depleting LLPCs, as this could compromise the patient’s immune defenses against infections (6, 72, 117). The balance between effective autoimmune disease management and preserving immune competence is crucial.

Targeting LLPCs in autoimmune diseases through various therapeutic approaches shows promise but requires careful consideration of the broader immunological impact, especially concerning infection risks and the maintenance of protective immunity. Further research and clinical trials will help refine these strategies to optimize safety and efficacy.

## 8 Selective targeting of autoreactive B cells

Selective targeting of autoreactive B cells through CAAR T cell therapy represents a significant advancement in treating autoantibody-driven autoimmune diseases (65). This approach is designed to enhance specificity in targeting pathogenic B cells, potentially reducing off-target effects and sparing nonpathogenic B cells, which are essential for normal immune function (118).

CAAR T cells are engineered to express pathogenic autoantigens as the extracellular domain of a chimeric immunoreceptor, allowing them to specifically target and eliminate B cells that recognize these autoantigens (65). This method provides a more selective approach compared to broad B cell depletion strategies like those involving anti-CD19 CAR T cells.

The effectiveness of rituximab in treating pemphigus vulgaris by reducing anti-desmoglein (DSG) antibodies highlights the pivotal role of DSG-specific B cells in the disease (119, 120). CAAR T cells engineered to target DSG3-specific B cells have shown promising results in preclinical studies, expanding, persisting, and effectively depleting these autoreactive B cells (65). Clinical trials, such as the one registered under NCT04422912, are currently evaluating DSG3-CAAR T cells in patients with mucosal pemphigus vulgaris (4, 121).

In myasthenia gravis, autoantibodies against muscle-specific kinase (MuSK), essential for neuromuscular transmission, play a pathogenic role (122, 123). CAAR T cells that target MuSK-specific B cells have been developed, incorporating the MuSK ectodomain with CD137–CD3ζ signaling domains. Preclinical studies have demonstrated that MuSK-CAAR T cells can effectively reduce anti-MuSK antibody titers without the broad depletion of B cells typically seen with anti-CD19 CAR T cells (124–126). A clinical trial (NCT05451212) is underway to test MuSK-CAAR T cells in patients with anti-MuSK antibody-associated myasthenia gravis (112, 127).

The CAAR T cell approach is particularly advantageous for diseases with a well-characterized autoantigen and a clearly defined pathogenic role of specific autoantibodies. This therapy can potentially offer targeted treatment options that minimize unnecessary immune suppression and preserve the immune system’s capacity to respond to other antigens, thus maintaining better overall immune health (118).

CAAR T cell therapy holds significant promise for improving treatment specificity and efficacy in autoantibody-driven autoimmune diseases, offering a tailored approach that could enhance patient outcomes while minimizing collateral immune suppression. Continued research and clinical trials are crucial to establish the efficacy and safety of CAAR T cell therapies in various autoimmune conditions. These studies will help determine the long-term outcomes, optimal protocols for administration, and potential side effects of selectively targeting autoreactive B cells in a clinical setting.

## 9 Harnessing Tregs

Harnessing Tregs for therapeutic purposes is a promising strategy for treating autoimmune diseases, graft-versus-host disease (GVHD), and even in cancer settings to modulate immune responses and restore immune homeostasis (26, 27, 128).

Treg cells are known for their ability to suppress inflammation and autoimmunity through various mechanisms. They can engage in direct inhibitory interactions with other immune cells via cell surface receptors or indirectly by producing soluble inhibitory mediators like interleukin-10 (IL-10) and transforming growth factor-beta (TGF-β). These properties make Tregs a valuable component in the treatment of autoimmune disorders (129). Initial clinical investigations using *ex vivo* expanded polyclonal Treg cells have shown that they are well tolerated in patients with various autoimmune diseases and can persist in the circulation for up to a year after administration (28). However, the clinical impact of these polyclonal Treg therapies has been relatively modest, likely due to their non-specific nature (129–131).

By contrast, antigen-specific Treg cells, which are more targeted, have demonstrated greater potency and efficacy in preclinical models. These cells can be engineered to home to specific sites of inflammation or to interact with specific effector T cells, providing localized immune modulation with potentially reduced risks of broad immunosuppression (132–134). Recent advances include engineering Tregs with CARs or specific T-cell receptor (TCR) sequences to enhance their specificity and efficacy. For instance:

These are Treg cells modified with CARs to recognize specific antigens, such as CD19. This approach has been explored in models where anti-CD19 CAR Treg cells have been shown to suppress IgG antibody production and B cell differentiation through a TGFβ-dependent mechanism, suggesting a novel method to control autoantibody production without needing to fully deplete B cells (135, 136). By selecting specific TCR sequences, Tregs can be directed towards particular antigens or inflammation sites, enhancing their therapeutic potential in autoimmune and alloimmune conditions (136).

### 9.1 Mechanism of CAR Treg mediated suppression in autoimmune setting

CAR Tregs are adept at recognizing both cell-surface and soluble antigens, facilitating versatile immunoregulatory roles in autoimmune conditions. Upon encountering cells that express the target antigen on their surface, CAR Tregs are activated. This activation triggers the secretion of anti-inflammatory cytokines, including interleukin-10 (IL-10) and TGF-β, and the release of cytotoxic molecules such as perforin and granzyme (62, 137–139). Furthermore, CAR Tregs enhance the expression of co-inhibitory receptors like CTLA-4, which play a critical role in maintaining immune homeostasis by delivering inhibitory signals to effector T cells (140).

Additionally, CAR Tregs upregulate CD25, the alpha chain of the IL-2 receptor, which leads to increased consumption of IL-2 (27, 128, 141). This IL-2 consumption effectively deprives effector T cells of a critical growth factor, indirectly suppressing their proliferation and function. This mechanism is crucial in modulating immune responses in autoimmune diseases, where limiting the activation and expansion of pathogenic T cells can mitigate disease progression.

**Figure 4:**
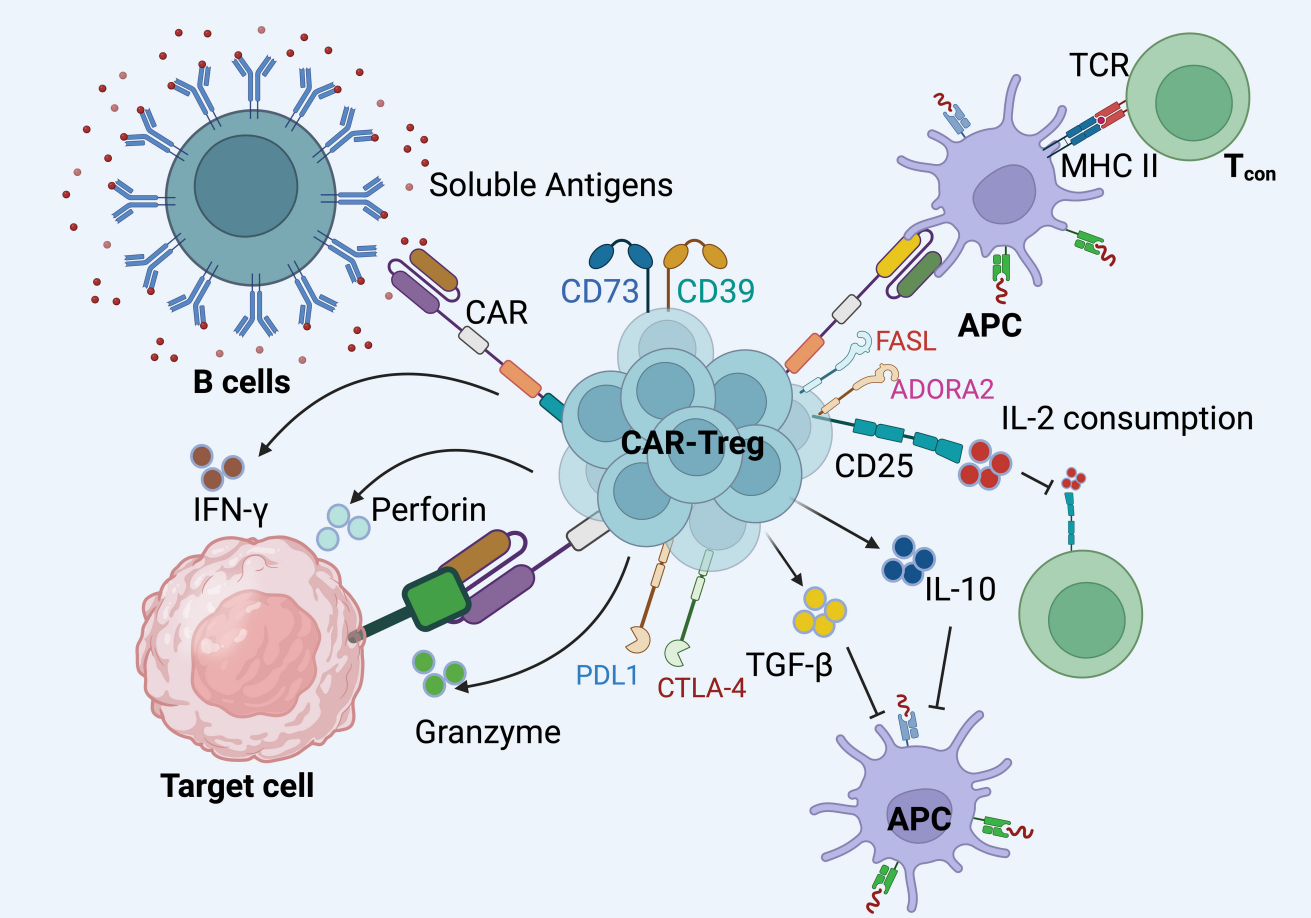
The mechanisms through which CAR Tregs exert their immunosuppressive effects in autoimmune settings. i). CAR Tregs are engineered to express specific chimeric antigen receptors that enable them to recognize both cell-surface and soluble antigens. These receptors facilitate targeted interactions with antigens presented by B cells or antigen-presenting cells (APCs). ii). Upon engagement with their target antigens, CAR Tregs initiate a cascade of immunosuppressive responses. They secrete anti-inflammatory cytokines such as IL-10 and TGF-β, which help modulate the inflammatory environment. Furthermore, CAR Tregs can release cytotoxic molecules like perforin and granzyme to directly suppress target cells. iii). These CAR Tregs cells also upregulate co-inhibitory molecules such as CTLA-4 and PD-L1, which contribute to the inhibition of effector T cell (Tconv) function. CTLA-4, in particular, competes with Tconv cells for CD80/CD86 on APCs, thereby inhibiting their activation.iv). CAR Tregs express high levels of CD25, the IL-2 receptor alpha chain, leading to increased consumption of IL-2. This deprives Tconv cells of a critical growth factor, further suppressing their proliferation and activity. v). Interactions between Tconv cells and APCs can also be modulated by CAR Tregs through their immunosuppressive actions, potentially altering the outcome of Tconv cell responses. vi).The interaction of CAR Tregs with FAS ligand (FASL) and adenosine A2A receptor (ADORA2), which are additional pathways through which these cells can exert regulatory effects, further enhances their ability to control autoimmune responses. Created in BioRender.com.

In scenarios involving soluble antigens, CAR Tregs can recognize antigens that are non-specifically bound to the surfaces of APCs or to B cell receptors (BCR) on B cells. This interaction initiates a cascade of both contact-dependent and -independent immune suppression. Contact-dependent mechanisms may involve direct cell-cell interactions that engage inhibitory pathways, while contact-independent mechanisms likely involve the secretion of soluble factors that modulate immune responses in the local microenvironment. Although these processes are pivotal in regulating autoimmune responses, the exact mechanisms by which CAR Tregs exert their suppressive effects in the presence of soluble antigens are still being elucidated, indicating a rich area for ongoing research (**Figure 4**).

The application of CAR Treg cells has shown promising results in controlling autoimmune diseases and preventing GVHD in transplantation contexts without eliminating beneficial immune responses (142). For example, anti-CD19 CAR Treg cells have not only modulated B cell functions in autoimmune models but also mitigated acute GVHD while preserving graft-versus-tumor effects in cancer models (135, 143, 144) (**Table 2**). Furthermore, incidental findings from leukemia treatments have highlighted the presence of non-clonal CD4+ Helios+ CAR T cells post-infusion, which exhibit characteristics of Tregs and are associated with less severe neurotoxicity and disease progression (158).

**Table 2:** Possible application of CAR-Tregs for various diseases.

Disease Model	Antigen Specificity	Functional Characteristics	Region	Institution	Organizer	Reference
Autoimmune Disease						
Colitis	TNP	Reduction of mouse colitis by CAR-Treg. More effective than polyclonal Tregs	Israel	Weizmann Institute of Science	Elinav E	(145, 146)
Colitis and colorectal cancer	CEA	More effective than irrelevant CAR-Tregs in improving colitis and colitis-related colorectal cancer	Israel	Weizmann Institute of Science	Blat D	(147)
MS	MOG	Naive CD4+ T cells turned into Tregs by increasing FOXP3 expression Reducing EAE more effectively than MOCK-transduced Tregs	Sweden	Uppsala University	Fransson M	(148)
Transplantation						
GVHD	HLA-A2	Better than polyclonal Tregs at stopping xenogeneic GVHD after human PBMCs are transplanted	Canada	University of British Columbia	MacDonald KG	(149)
Skin Transplant Rejection	HLA-A2	Completely stopping the rejection of HLA-A2-positive blood cells and skin transplants	German	Hannover Medical School	Noyan F	(150)
Skin Transplant Rejection	HLA-A2	Stopping the rejection of human HLA-A2-positive skin grafts more effectively than polyclonal Tregs.	UK	King’s College London	Boardman DA	(151)
GVHD, Islet,and Skin transplantation	Universal	Activation of mAbCAR-Treg by FITC-labeled mAb. Stopping GVHD and increasing the survival of islet and secondary skin transplants.	USA	Stanford University School of Medicine	Pierini A	(152)
MISCELLANEOUS						
Hemophilia A	FVIII	CAR-Treg activated by soluble protein FVII. Suppression of anti-FVIll antibody responses	German	Uniformed Services University of Health Sciences	Yoon J	(153)
Asthma	CEA	More effective at controlling asthma than unmodified Tregs	German	Hannover Medical School	Skujec J	(154)
Burkitt lymphoma	CD19	Reducing the antitumor effectiveness of CD19-specific CAR-T	USA	Memorial Sloan-Kettering Cancer Center	Lee JC	(155)
Sarcoma	CEA	Reducing the antitumor effectiveness of CD19-specific CAR-T	German	Universität zu Köln, Köln	Hombach	(156)
Prostate cancer	Universal	Activation of UniCAR-Treg by a peptide E5B9-linked mAb/scFv aimed at a cell surface structure Costimulation with CD137 is safer than with CD28 Costimulation with CD137 is safer than with CD28	German	Institute of Radiopharmaceutical Cancer Research	Koristka S	(157)

GVHD, graft-vs.-host disease; HLA, human leukocyte antigen; PBMC, peripheral blood mononuclear cell; mAb, monoclonal antibody; FITC, fluorescein isothiocyanate; TNP, 2,4,6- trinitrophenol; CEA, carcinoembryonic antigen; MOG, myelin oligodendrocyte glycoprotein; EAE, experimental autoimmune encephalomyelitis; FVIII, Factor VIII; scFv, single chain variable fragment.

Harnessing CAR Treg cells offers a sophisticated approach to modulating immune responses, particularly in the context of autoimmune diseases, organ transplant rejection, and inflammatory conditions. The development and refinement of Treg-based therapies represent a significant advance in immunotherapy, offering a nuanced approach to treating a variety of immune-mediated diseases. The advantages and challenges associated with CAR Treg cells stem from their unique properties and the complexities of their engineering and application.

### 9.2 Safety considerations for CAR Treg cells

Unlike conventional CAR T cells, CAR Treg cells pose a minimal risk for CRS because they inherently do not produce pro-inflammatory cytokines (25, 159). A critical safety concern with CAR Treg cells is ensuring the stability of the Treg phenotype. The risk that Treg cells could revert to an effector T cell phenotype (ex-Treg cells) is a debated but recognized concern (160–162). Stability is typically associated with persistent expression of the transcription factor FOXP3 and specific epigenetic markers such as demethylation in the Treg-specific demethylation region (160, 163).

To enhance the stability of CAR Treg cells, strategies include using strong promoters to drive the expression of FOXP3 or employing gene-editing techniques to ectopically express FOXP3. These methods aim to maintain the suppressive characteristics of Treg cells and prevent their conversion into potentially harmful effector T cells.

### 9.3 Applications of CAR Treg cells

**Figure 5:**
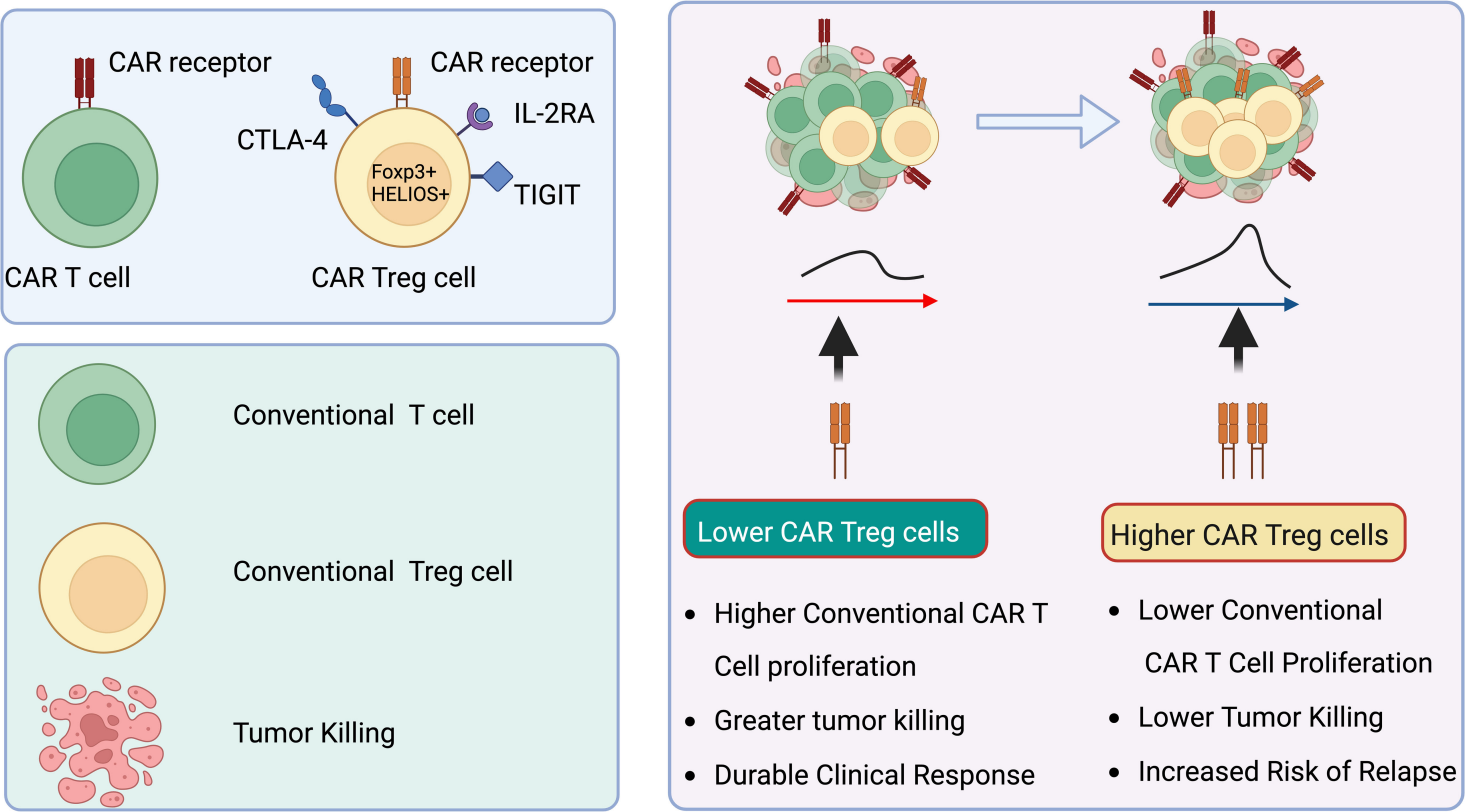
CAR Treg cells correlate with reduced efficacy of anti-CD19 CAR T cell therapy in lymphoma patients. CAR Treg cells are linked to suboptimal outcomes in lymphoma patients treated with anti-CD19 CAR T cell therapy. Elevated levels of CAR Treg cells within CAR products, along with their growth post-infusion, inhibit the proliferation and activity of standard cytotoxic CAR T cells. This suppression results in enhanced tumor expansion. Created in BioRender.com.

In an insightful exploration of the molecular dynamics within CAR T cell therapies, recent studies have elucidated the pivotal role of CAR-Treg in influencing the therapeutic outcomes of treatments for B cell malignancies (158, 164). According to Haradhvala et al. and Good et al. in their publications in Nature Medicine, the presence and modulation of CAR-Treg cells within the therapeutic products, such as axicabtagene ciloleucel (axi-cel) and tisagenlecleucel (tisa-cel), have been closely linked to patient responses and therapeutic resistance (165). These studies employed cutting-edge single-cell transcriptomic and proteomic profiling to dissect the variations in cellular phenotypes post-infusion and correlated these with clinical outcomes. Significantly, the findings suggest that subtle differences in manufacturing processes and CAR construct design, particularly concerning the inclusion or exclusion of Tregs, may critically impact the efficacy and safety profiles of these treatments. These insights are instrumental for refining CAR T cell therapy strategies, emphasizing the need for targeted manipulation of CAR-Treg cells to potentially enhance clinical responses and mitigate relapse rates in lymphoma treatments (**Figure 5**).

#### 9.3.1 T1DM

**Figure 6:**
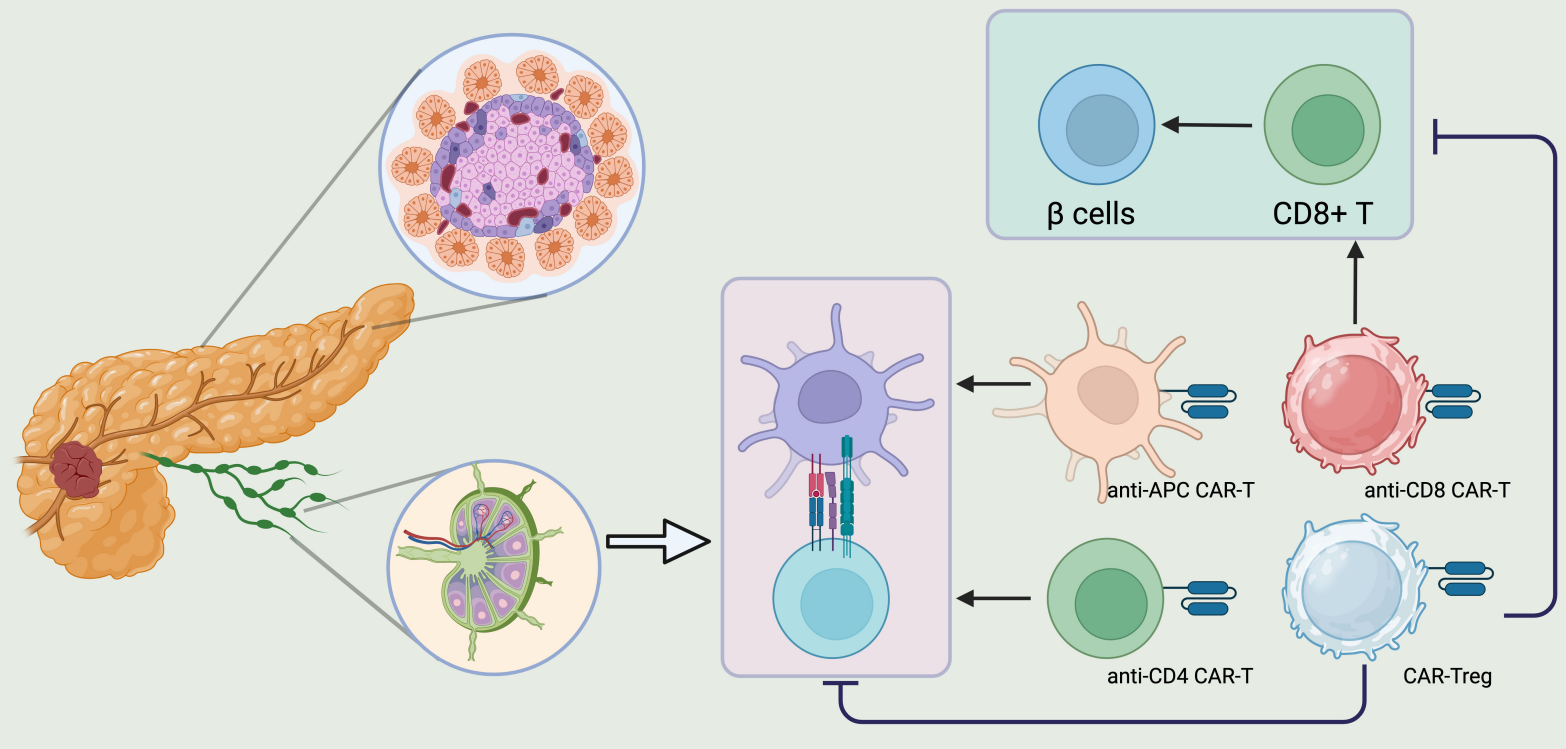
CAR T regs could be a promising therapeutic strategy to reverse the course of T1DM. CD8+ T cells, CD4+ T cells, APCs, and Tregs play a crucial role in the development of T1DM. Consequently, creating CARs targeting CD4, CD8, and APCs could be a promising strategy to protect pancreatic beta cells from destruction. On the other hand, Treg deficiency leads to a breakdown in peripheral immune tolerance, triggering cytotoxic T cell activation and the production of autoantibodies against pancreatic beta cells. Therefore, engineering CAR Tregs to address Treg insufficiency in T1DM patients and suppress the autoreactive immune system could be an effective therapeutic approach to alter the disease’s progression. Created in BioRender.com.

T1DM is an autoimmune disorder characterized by the destruction of insulin-secreting pancreatic β-cells. While insulin replacement therapy remains the primary treatment for T1DM, it does not halt the disease’s progression, as ongoing immune dysregulation and inflammation continue to damage pancreatic β-cells (166). Consequently, adopting immunomodulatory therapies could be beneficial in preventing and reversing the disease’s advancement. Various immune-modulatory approaches, such as mAb-based therapies, mesenchymal stem cell treatments, and immune cell therapies, could be considered (167, 168). However, because these approaches may impact the entire immune system and potentially cause toxicity, more targeted treatments that specifically address the immune response against pancreatic β-cells are needed. In this context, CAR-based immunotherapy emerges as a promising candidate for correcting dysregulated immune responses in T1DM (4, 169). CAR-based therapy has already shown effectiveness in treating several hematologic cancers (170). Recently, there has been renewed interest in applying CAR T cells as a “ready-to-use” treatment for T1DM (171). Preclinical studies have shown that directing antigen-specific CAR T cells, particularly CAR Tregs, towards pancreatic β-cells could prevent or slow the development of diabetes in mouse models (172) (**Figure 6**).

#### 9.3.2 Inflammatory bowel disease

CAR-Treg technology has made considerable progress, yet the challenge of expanding Treg cells *in vitro* to adequate numbers persists. Research across various disease models has shown that localized delivery or antigen-specific Treg transfer can enhance immunosuppression with fewer cells. It has been suggested that CAR engineering, initially developed for directing effector T cells to tumors, could be adapted for targeting Tregs. Indeed, a recent study involving patients with large B-cell lymphoma demonstrated that the frequency of CAR Tregs within transferred CD19-CAR T cells inversely correlated with CAR T cell expansion and disease progression, while an increase in CAR Tregs was associated with a reduced risk of neurotoxicity (158). CAR expression in Treg cells via lentiviral delivery has been successfully achieved in both mouse and human Tregs (173). Additionally, the CD28 costimulatory domain has been shown to maintain the suppressive function of CAR-Tregs more effectively than the 4-1BB domain in both *in vitro* and *in vivo* settings (174–176).

CAR-Tregs have been effective in suppressing inflammation and reducing disease severity in mouse models, including experimental autoimmune encephalomyelitis (EAE) and GVHD (175–178). This promising preclinical data has led to the initiation of the first CAR-Treg clinical trial, using HLA-A2-specific CAR Tregs in kidney transplant patients (EUCTR2019-001730-34-NL) (179). However, in some disease models, even though antigen-specific Tregs showed stable, suppressive, and long-lived characteristics, such as insulin-specific CAR Tregs, they did not prevent disease development, as observed in diabetes models (176).

In the context of inflammatory bowel disease (IBD), there is substantial interest in utilizing Treg cells to modulate inflammation. This strategy has been validated in T cell transfer and AOM-DSS models of colitis, targeting carcinoembryonic antigen (CEA), which is elevated in the gastrointestinal tract in both colorectal cancer and colitis. Systemically administered CEA-specific CAR Tregs accumulated in the colons of CEA transgenic mice, leading to a reduction in colitis severity and subsequent tumor development. Similarly, transgenic mice expressing a 2,4,6-Trinitrophenol (TNP)-specific CAR were resistant to TNBS-induced colitis (145). Treg cells from these transgenic mice, or from wild-type mice transduced with the CAR, exhibited antigen-specific suppression *in vitro*, and their transfer led to accumulation at inflamed colonic sites, resulting in antigen-specific disease reduction in TNBS-induced colitis (but not in oxazolone-induced colitis) (146). Further research showed that TNP-CAR Tregs exerted their suppressive effects through a contact-dependent mechanism, largely independent of IL-10 or TGF-β. Despite successful targeting in murine colitis models, identifying appropriate antigens for targeting CAR Tregs to the intestines in human IBD patients remains an ongoing challenge (146).

#### 9.3.3 Multiple sclerosis

CAR therapies for MS have predominantly focused on the development of CAR-Tregs. In a study by Fransson et al., CD4+ T cells were transduced using a lentiviral vector system to express a CAR targeting myelin oligodendrocyte glycoprotein (MOG) (148). These cells were then induced to differentiate into Tregs through the expression of mouse FoxP3 and incorporation of CD28-CD3ζ signaling domains. Both *in vitro* and *in vivo* experiments demonstrated that CARαMOG-FoxP3-Tregs effectively suppressed T cell proliferation in the presence of MOG+ cells and activated macrophages (148). For *in vivo* studies, these cells were administered intranasally to mice with experimental autoimmune encephalomyelitis (EAE). The findings showed that 24 hours after infusion of GFP and CARαMOG-FoxP3-co-expressing Tregs, markers of myelination (myelin basic protein, MBP) and reactive astrogliosis (glial fibrillary acidic protein, GFAP) were restored in treated mice compared to controls, indicating successful targeting by CAR-Tregs (148). Moreover, mice with an EAE score of 4 (indicating hind limb paralysis) showed a reduction in disease severity in both the CD4+ T cell and CAR-Treg groups. By day 7, only the CAR-Treg group exhibited continuous symptom relief, and by day 25, the CAR-Treg-treated mice were asymptomatic. When rechallenged with a second EAE-inducing inoculum, these mice remained healthy, demonstrating the long-lasting effect of the engineered Tregs. Additionally, the CAR-Treg group showed decreased expression of IL-12 and IFN-γ mRNA in the brain, likely due to reduced inflammation and inhibition of dendritic cell maturation (148).

In 2020, leveraging the bystander effect, researchers explored the combination of MBP-CAR Treg and MOG-CAR Treg therapies in an EAE model, discovering that this dual approach significantly suppressed inflammation and myelin-specific T cell responses, thereby slowing EAE progression (177).

The benefits of CAR therapy for MS are evident. First, intranasal delivery allows for targeted accumulation of CAR-Tregs at the site of interest with minimal dosage, reducing the risk of systemic exposure and off-target effects in other vital organs. Second, CAR-Tregs demonstrate strong specificity and durability; for instance, they can still prevent disease progression upon re-exposure to EAE antigens. However, safety concerns remain. The highly vascularized nature of the nasal mucosa raises the possibility that CAR-Tregs could enter systemic circulation. Additionally, post-nasal administration, cells could potentially migrate from the nasal mucosa to the brain and cerebrospinal fluid (CSF) via the olfactory nerve pathway (180).

#### 9.3.4 Systemic lupus erythematosus

**Figure 7:**
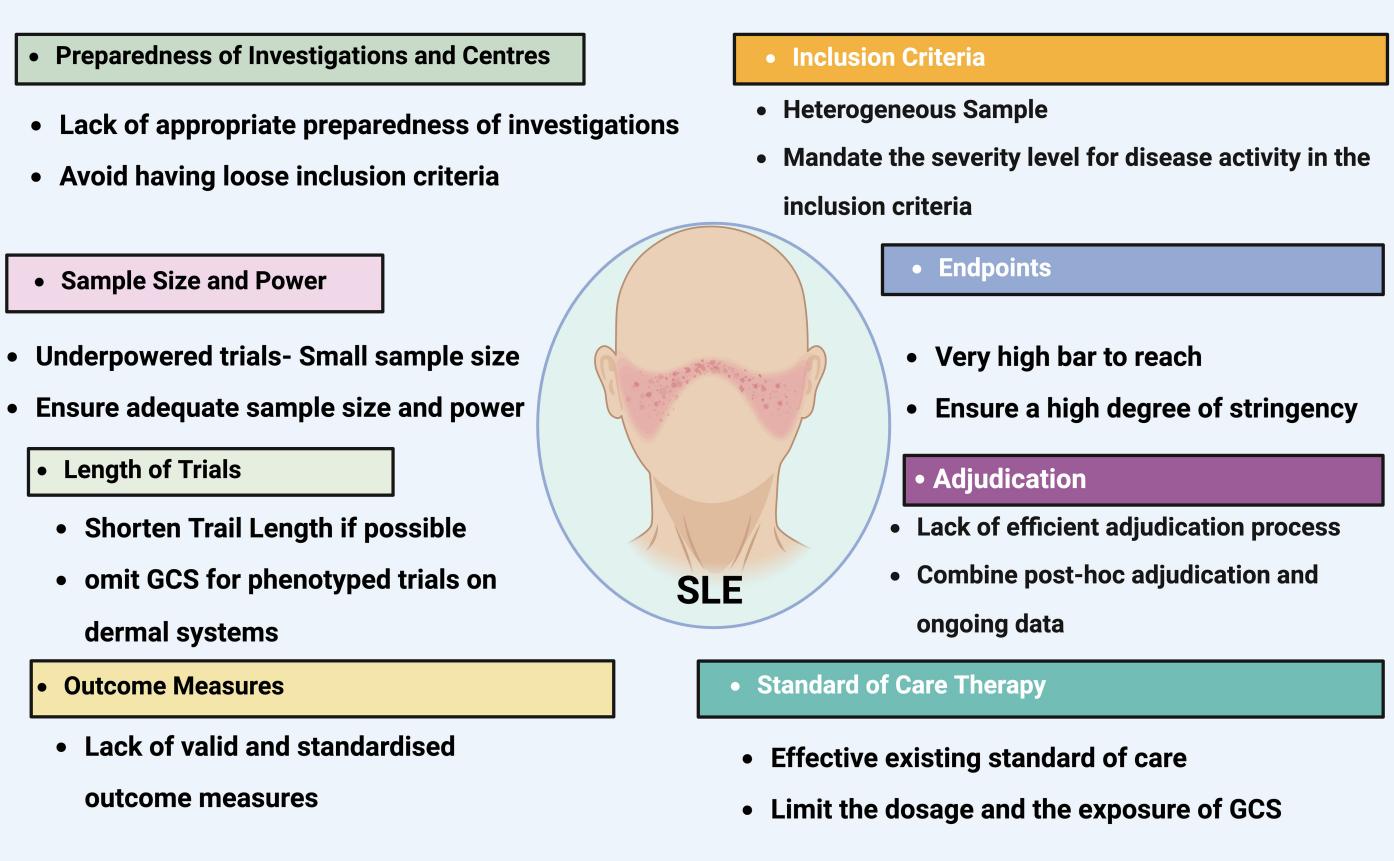
The most common pitfalls in lupus clinical trials. Titles on colored background identifies the most frequent issues that have impeded the effectiveness of lupus clinical trials. The contents detail the areas where these issues commonly arise, provide a deeper understanding of each challenge, and offer some recommendations for addressing these concerns. *GCS, glucocorticosteroid*. Created in BioRender.com.

SLE is a multifaceted autoimmune disorder characterized by a failure in immunologic self-tolerance (181). The pathogenesis of SLE is driven by autoreactive B and T cells, along with key components of the innate immune system (182, 183). Numerous therapeutic strategies have been explored, many showing promise in modifying the disease course by controlling symptoms and slowing progression, though none offer a cure (184) (**Figure 7**). Recent advancements in understanding SLE pathogenesis have led to the development of more targeted therapies, particularly mAbs that focus on disease-specific molecules (185). However, these biologic therapies often require continuous administration to manage disease manifestations and carry a cumulative risk of infections and other comorbidities.

A significant challenge in achieving long-term remission in SLE is the presence of autoreactive immunologic memory, which typically forms well before the onset of symptoms and is largely resistant to current biologic therapies, especially those targeting memory plasma cells that secrete autoantibodies (186, 187). To address this, two distinct therapeutic approaches have been developed: one focuses on eliminating autoreactive immune cells (immunoablation), and the other aims to restore immune tolerance (immune regulation).

For immunoablation, autologous hematopoietic stem cell transplantation (HSCT), and less commonly allogeneic HSCT, has been employed in SLE patients, providing proof-of-concept that long-term remissions can be achieved by resetting the immune system to a self-tolerant state. However, autologous HSCT is associated with considerable risks, including transplant-related mortality and long-term complications such as secondary autoimmune diseases (187). It remains uncertain which specific components of the memory immune compartment should be targeted and the extent of lymphocyte lineage depletion required for sustainable remission in SLE. Ongoing trials involving CD19 CAR-T-cell therapies in SLE are of particular interest, as they offer a broader depletion of autoreactive B cells, including those residing in inflamed tissues (5, 19). Preliminary results suggest that this approach can achieve significant B cell depletion and reductions in autoantibodies, but it is still unclear if this is sufficient for durable remission, or if additional memory compartments, such as CD19-negative plasma cells or T cells, need to be targeted.

Another therapeutic strategy for managing chronic autoimmune responses in SLE involves enhancing immune regulation to restore self-tolerance. Tregs, which play a crucial role in maintaining self-tolerance, are central to this approach. Various strategies are under investigation, including methods to directly augment Treg cells *in vivo*, such as with IL-2 or IL-2 muteins, or through ex vivo manipulation followed by reinfusion of Treg cells (188–190). Clinical trials of adoptive Treg-cell therapies have demonstrated feasibility and initial efficacy. However, the use of polyclonal Treg cells has yielded only modest and transient clinical benefits, likely due to the limited number of disease-relevant Treg cells in the therapeutic product or their reduced persistence *in vivo* (191). To address these limitations, new approaches involving engineered regulatory cells are being explored, which may offer a more effective solution for inducing sustained immune tolerance in SLE (159).

#### 9.3.5 Organ transplantation

The MHC class I molecule is constitutively expressed on nearly all transplanted cells, including passenger leukocytes that migrate from a graft. Notably, HLA-A2 is highly prevalent, found in over 40% of white donors (192, 193). Moreover, mismatching of HLA-A is often linked to poor transplantation outcomes, making HLA-A2 a promising target antigen for generating antigen-specific Tregs to induce transplantation tolerance.

Alloantigen-specific human Tregs were engineered with an HLA-A2-specific CAR (A2-CAR) in a peptide-independent manner and utilized to prevent xenogeneic graft-versus-host disease (GVHD) in immunodeficient NOD.SCID.γc-/- (NSG) mice that received HLA-A2+ human peripheral blood mononuclear cells either alone or in combination with A2-CAR-expressing Tregs (149). The A2-CAR Tregs maintained high expression levels of canonical Treg markers, including FoxP3, CD25, Helios, CTLA-4, along with a high degree of demethylation in the Treg-specific demethylated region of the FOXP3 locus. The A2-CAR provided stronger antigen-specific activation in Tregs compared to the endogenous TCR. When activated via CARs, A2-CAR Tregs suppressed CD8+ T cell proliferation *in vitro* and were more effective than TCR-Tregs in preventing xenogeneic GVHD in NSG recipients. Unlike TCRs, CARs also stimulated IL-2-independent Treg proliferation in the short term. Moreover, CAR-stimulated Tregs exhibited higher surface expression of CTLA-4, latency-associated peptide (LAP), and the inactive precursor of TGF-β, suggesting that CAR-Tregs may offer advantages over TCR-Tregs (149).

Subsequently, A2-CAR-Tregs were developed to prevent skin allograft rejection (150). The A2-CAR altered the specificity of natural Tregs (nTregs) without changing their regulatory phenotype or epigenetic stability. Activation through the A2-CAR led to stronger cell proliferation, upregulation of the effector molecule CD39, and more effective inhibition of allospecific effector T (Teff) cell proliferation *in vitro* compared to unmodified nTregs or control CAR-Tregs. Furthermore, in experiments measuring ear thickness in NOD.Rag1nullIL-2γc (NRG) mice receiving A2-CAR Tregs or control nTregs plus HLA-A1+ PBMCs (responders) and irradiated HLA-A2+ PBMCs (stimulators), A2-CAR Tregs more potently suppressed the allogeneic response of delayed-type hypersensitivity compared to unmodified nTregs or control CAR-Tregs (150). While adoptive transfer of polyclonal nTregs or control CAR-Tregs had only a moderate effect on inducing tolerance, A2-CAR-Tregs completely prevented the killing of allogeneic HLA-A2-positive target cells and rejection of HLA-A2-positive human skin grafts for over 40 days. Histological examination revealed that transferred A2-CAR-Tregs homed to skin grafts and persisted long-term (150).

Meanwhile, a similar study was conducted with two engineered HLA-A2-specific CARs: one with a CD28-CD3ζ signaling domain (CAR) and another lacking an intracellular signaling domain (ΔCAR) (151). This study found that, compared to polyclonal Tregs, both CAR and ΔCAR Tregs migrated through HLA-A2+ endothelial monolayers much faster than through HLA-A2− endothelial monolayers *in vitro*, confirming the preferential migration of A2-CAR-Tregs into HLA-A2+ target tissues (150). This suggests that the expression of target antigens on transplanted cells would likely stimulate the localization of antigen-specific CAR-Tregs to a graft. Indeed, Treg localization to the graft is crucial for preventing allograft rejection and inducing transplant tolerance (194). These studies on A2-CAR Tregs imply a promising clinical application for CAR-Treg therapies.

*Antonio* et al. developed an innovative CAR, termed mAbCAR, which incorporates a FITC-targeted CAR on Tregs (152). This design allows for flexible activation using various mAbs that are covalently linked to FITC. Their study demonstrated that mAbCAR Tregs can indeed be activated by FITC-conjugated antibodies, and these antigen-specific mAbCAR Tregs retained their original phenotypes and functional characteristics. In an experimental model, the adoptive transfer of donor-derived MAdCAM1-mAbCAR Tregs into lethally irradiated allogeneic BALB/c mice, prior to the administration of allogeneic donor T cells and T cell–depleted bone marrow, effectively prevented GVHD. Furthermore, when comparing donor-specific H-2Dd-mAbCAR Tregs, which target the MHC-I antigen H-2Dd expressed on transplanted islets, with isotype-mAbCAR Tregs, the former significantly prolonged islet allograft survival (152).

Bioluminescent imaging and histological analysis revealed that H-2Dd-mAbCAR Tregs exhibited an enhanced capacity to home to and expand within the islet grafts. Additionally, they demonstrated that these Tregs induced alloantigen-specific peripheral tolerance, as evidenced by the prolonged survival of secondary skin allografts in mice treated with H-2Dd-mAbCAR Tregs. This study indicates that the antigen-specific activation of mAbCAR Tregs, achievable by various FITC-conjugated mAbs targeting different surface proteins, enhances their ability to home to specific tissues, thus suggesting a potential application of mAbCAR technology in Treg-based therapies. Moreover, a similar flexible CAR module, known as universal CAR (UniCAR), has recently been introduced, as detailed in discussions on CAR-Tregs for other diseases (157).

#### 9.3.6 Other types of autoimmune diseases

Patients with other types of autoimmune disorders, including idiopathic inflammatory myopathy, systemic sclerosis, neuromyelitis optica spectrum disorder and myasthenia gravis, have also undergone CAR T cell treatments targeting B cells or plasma cells (4, 5, 10, 12–16, 22, 23, 102, 117, 195–200). The central role of B cells and the formation of autoantibodies in the pathogenesis of these diseases necessitates profound B cell depletion, including the elimination of autoreactive B cell clones, for the effectiveness of CAR T cell therapies that utilize anti-CD19 or anti-BCMA targets. These therapies are therefore predominantly suitable for conditions significantly driven by B cell activity, where the presence of pathogenic autoantibodies indicates a susceptibility to antibody-mediated B cell depletion. In contrast, chronic inflammatory diseases such as psoriasis, inflammatory bowel disease, and spondylarthritis, which lack a significant pathogenic B cell role and are characterized instead by aberrant T cell activation and IL-23–IL-17-mediated inflammation, are less likely to benefit from B cell-targeted CAR T cell strategies (201, 202). These conditions might instead respond better to alternative cell-based therapies, including transfers of Tregs or mesenchymal stromal cells. However, other rheumatologic conditions such as granulomatosis with polyangiitis, rheumatoid arthritis, and primary Sjögren’s syndrome do exhibit considerable B cell involvement in their pathogenesis and might be suitable candidates for B cell-directed CAR T cell therapies (98, 203). Additionally, autoimmune diseases outside rheumatology like pemphigus vulgaris and primary sclerosing cholangitis may also be promising targets for this form of treatment (66, 204).

The successful evaluation of CAR T cell therapies, particularly in controlled trials, hinges not only on the pathophysiology of the targeted disease but also on the presence of measurable and reliable clinical endpoints to assess therapeutic success. For example, evaluating therapeutic effects in diseases such as primary Sjögren’s syndrome is exceptionally challenging due to its complex clinical manifestations (205). This complexity has slowed the systematic exploration of CAR T cell therapy for primary Sjögren’s syndrome, despite its pronounced B cell activation, which can even lead to malignant transformations such as lymphoma (206). Nonetheless, there are isolated cases where patients with concurrent Sjögren’s syndrome and cancer showed improvement in both the cancer and symptoms of Sjögren’s syndrome following CAR T cell therapy (11). Similar challenges are encountered in certain forms of MS, where demonstrating a reduction or cessation of disease flares to establish therapeutic efficacy could take years, making such long-term studies particularly demanding.

Selecting appropriate co-stimulatory domains is crucial for optimizing CAR Treg cell function. While CD28 has been shown to play a significant role in Treg cell development and function, there is evidence suggesting that CAR Treg cells incorporating the 4-1BB co-stimulatory domain may have less stable lineage properties and reduced suppressive capacity compared to those containing CD28 (207). This difference highlights the nuanced roles of co-stimulatory domains in maintaining the functionality and stability of CAR Treg cells. Furthermore, integrating additional genetic modifications, such as chemokine receptors or cytokines (e.g., IL-10 or amphiregulin), can enhance the homing, immunosuppressive, and tissue-healing capacities of CAR Treg cells (32, 208). These modifications aim to create a more robust and targeted therapeutic profile, potentially improving efficacy in specific disease contexts. The development and application of CAR Treg cells represent a cutting-edge advance in immunotherapy, offering the potential to precisely modulate immune responses with fewer side effects than broad-acting immunosuppressants. Continued research and clinical trials will be essential to fully understand the capabilities, limitations, and long-term implications of this promising therapeutic strategy.

## 10 Conditioning chemotherapy considerations

In cancer therapy, conditioning chemotherapy is usually required before CAR T cell therapy to enhance the effectiveness of the infused T cells (209, 210). This preparation helps by depleting Tregs, increasing the availability of cytokines like IL-15 and IL-7, and possibly reducing myeloid-derived suppressor cells (211–213). Various chemotherapy regimens are utilized, including cyclophosphamide alone, a combination of cyclophosphamide and fludarabine, or bendamustine (109, 210, 214). The cyclophosphamide-fludarabine mix is often preferred as it has been shown to improve CAR T cell expansion and clinical outcomes better than cyclophosphamide alone (107, 108, 215, 216).

For autoimmune diseases, however, the ideal conditioning regimen remains uncertain (15). Cyclophosphamide-fludarabine has been used and is generally well-tolerated, commonly causing mild nausea, fatigue, and cytopenias. However, cyclophosphamide is associated with risks like secondary myeloid malignancies and potential fertility issues, especially concerning for the typically younger, female demographic affected by autoimmune diseases. Fludarabine has also been linked to rare cases of vision loss (20, 217–219). Although bendamustine has not been used for autoimmunity, it is shown promise in oncology by providing similar CAR T cell expansion without the neutropenia often seen with cyclophosphamide-fludarabine, although its side effects are comparable (220, 221).

Given these considerations, there is a growing need to explore less toxic conditioning options for autoimmune diseases due to different risk-benefit profiles compared to cancer treatments. While fludarabine might deplete beneficial Treg cells, cyclophosphamide could positively impact these cells by selectively depleting alloreactive T cells and sparing Treg cells, which might help establish early tolerance (222, 223). Given that Treg cells rely heavily on IL-2 and less on cytokines that increase post-lymphodepletion, conventional lymphodepleting regimens might not be necessary before CAR Treg cell therapy, pending further clinical developments (159, 224).

### 10.1 Concomitant immunosuppressive medications

Immunosuppressive medications routinely used in chronic autoimmune diseases could potentially impact the manufacturing and function of CAR T cells, necessitating their reduction or discontinuation prior to cell collection (apheresis) and infusion. In cases of active disease, completely eliminating these medications may not be feasible; thus, low doses of steroids (such as 10 mg/day of prednisone or its equivalent) may be utilized. The compatibility of higher doses of glucocorticoids with effective CAR T cell function remains unclear, though recent studies suggest certain steroids may be compatible without adversely affecting the anti-tumor capabilities of CAR T cells (225, 226).

Treg cells, which are critical in maintaining immune tolerance, are particularly vulnerable to antiproliferative drugs and calcineurin inhibitors (227). The extent to which background immunosuppression needs to be reduced or withdrawn when administering CAR Treg cells is still under investigation. Insights from their use in solid organ transplantation may guide the tapering process (224).

## 11 Potential for resetting the immune system and inducing enduring tolerance

The concept of an immunological ‘reset’ through comprehensive elimination of disease-driving B cells via CAR T cell therapies has been proposed. This approach might allow the immune system to reset itself, a phenomenon observed in the context of broad leukocyte depletion following hematopoietic stem cell transplantation (228). Despite the regeneration of B cells, sustained disease remission does not seem to require ongoing B cell aplasia. For instance, in a study involving patients with SLE treated with anti-CD19 CAR T cell therapy, naive B cells returned within about 110 days, yet no disease recurrence was reported up to a year after treatment. This is in contrast to cancer settings where B cell recovery can take much longer (22).

The persistence and long-term effects of the co-stimulatory domains (like CD28 or 4-1BB) within CAR constructs on T cell persistence and B cell aplasia duration remain to be fully understood (47). However, the initial depth of response in autoimmune contexts, as in oncology, might determine long-term treatment efficacy (229). The durability of tolerance induced by CAR Treg cell therapy and the maintenance of clinical benefits might depend on the long-term persistence and functional stability of the engineered cells at inflammation sites. In transplantation, continuous exposure to alloantigens may perpetuate the activation and suppressive functions of CAR Treg cells. Conversely, in autoimmune diseases, the reduction in inflammation might decrease both the number and activity of Treg cells over time. Nonetheless, studies have shown that even after CAR Treg cells are eliminated, reduced immune reactivity to specific antigens may persist. For example, adoptive transfer of human CAR Treg cells targeted against factor VIII in mice reduced immune reactivity against factor VIII for weeks after the CAR Treg cells were cleared, indicating the induction of a sustained tolerogenic state (153). This suggests that even transiently expressed CAR Treg cells could extend the survival of allogeneic transplants and promote long-term immune tolerance.

## 12 Conclusion

CAR T cells offer a more comprehensive depletion of tissue-resident B cells compared to mAbs, while CAR Treg cell therapies have the capability to sustain peripheral tolerance at inflammation sites. These advanced cellular therapies could potentially provide long-lasting remissions for various autoimmune diseases, marking a significant improvement over traditional treatments. Additionally, these targeted methods are likely to present a more favorable safety profile, which has been a challenge for previous cellular therapies used in autoimmunity, such as hematopoietic stem cell transplantation (230–232). This could make CAR T and CAR Treg cell therapies more broadly acceptable and safer alternatives for treating autoimmune conditions.
